# 

**Published:** 1869-04-15

**Authors:** 


### CONTENTS.

COMMUNICATIONS:

Discovery of a New Anatomical Feature in Human Blood Corpuscles....... 225

CORRESPONDENCE:

The Dynamics of Organic Life...... 232

Between Drs. Baldwin and Nott, in relation to the next meeting of the Am. Medical Association...... 257

TRANSLATIONS:

Medical Electricity...... 239

FOREIGN ITEMS:

Investigations into and Experiments upon the Physiological and Therapeutical Properties of Curare...... 252

LOOT:

L'Univers Medicale...... 254
Monstrosity...... 255
Fragilitas Ossium...... 255
Prolapsed Funis...... 256
Monstrosity...... 256

